# End-of-life decisions: how do patients die in the ICU?

**DOI:** 10.1186/cc14648

**Published:** 2015-03-16

**Authors:** S Barbosa, P Cavaleiro, J Guedes, S Castro, C Granja

**Affiliations:** 1 Unidade de Faro - Centro Hospitalar do Algarve, Faro, Portugal

## Introduction

One of the main goals of intensive care medicine is to reduce the mortality of critically ill patients. However, it is essential to recognize end-of-life care as an integral component of critical care. Besides survival, the success of intensive care should also include the quality of lives preserved and the quality of dying. The objective of this study was to evaluate the incidence and type of end-of-life decisions (ELD) in critical patients that died in an ICU.

## Methods

Analysis of all patients included in an ICU running database and who died from 1 November 2013 to 31 October 2014. The following variables were evaluated: age, gender, reason for admission, SAPS II, length of ICU stay and type of ELD. To classify ELD, four concepts were defined: 'Comfort care', a change from curative therapy to comfort care therapy; 'Limited therapy', maintenance of curative therapy but without escalating it (for example, no renal substitution); 'Decision not to resuscitate', not to perform advanced life support if cardiac arrest occurs; and 'Without previous end-of-life decisions', when there was no prior decision regarding the ELD.

## Results

A total of 507 patients were admitted to the ICU and 132 died (26%). Reasons for admission in those who died were septic shock (47%), post cardiac arrest (13%), cardiogenic shock (8%), and nontraumatic brain bleeding (8%). Fifty-three patients (40%) died after a 'Comfort care' decision, 28 patients (21%) after 'Decision not to resuscitate' and 14 (11%) after a 'Limited therapy' decision. Thirty-seven patients died 'Without previous end-of-life decisions'. However, specifically in this group, when looking for individual records, 32 patients died (86%) in the first 48 hours after the admission and four (11%) had evidence of brain death and were organ donors, which leaves one patient (3%) in whom there was no ELD.

## Conclusion

In this study, 'Comfort care' was the main ELD, which is in line with the concept that ELD are essential to ensure that care provided is consistent with quality of life and death. The apparent large proportion of patients 'Without previous end-of-life decisions' was due to patients who died in the first 48 hours after ICU admission corresponding to conditions refractory to treatment. Additionally, this study also draws our attention to better plan ICU admissions and hospital outreach in order to reduce early ICU mortality.

